# SLUG and SNAIL as Potential Immunohistochemical Biomarkers for Renal Cancer Staging and Survival

**DOI:** 10.3390/ijms241512245

**Published:** 2023-07-31

**Authors:** Maja Zivotic, Sanjin Kovacevic, Gorana Nikolic, Ana Mioljevic, Isidora Filipovic, Marija Djordjevic, Vladimir Jovicic, Nikola Topalovic, Kristina Ilic, Sanja Radojevic Skodric, Dusko Dundjerovic, Jelena Nesovic Ostojic

**Affiliations:** 1 Institute of Pathology, Faculty of Medicine, University of Belgrade, 1 Dr. Subotic Street, 11000 Belgrade, Serbia; maja.zivotic@med.bg.ac.rs (M.Z.); gorana.nikolic@med.bg.ac.rs (G.N.); ana.mioljevic@med.bg.ac.rs (A.M.); isidora.filipovic@med.bg.ac.rs (I.F.); kristina.ilic@med.bg.ac.rs (K.I.); sanja.radojevic-skodric@med.bg.ac.rs (S.R.S.); 2 Department of Pathological Physiology, Faculty of Medicine, University of Belgrade, 9 Dr. Subotic Street, 11000 Belgrade, Serbia; sanjin.kovacevic@med.bg.ac.rs; 3 Faculty of Organization Sciences, University of Belgrade, 11010 Belgrade, Serbia; md20115017@student.fon.bg.ac.rs; 4 Clinic for Cardiac Surgery, Clinical Center of Serbia, Faculty of Medicine, University of Belgrade, 11000 Belgrade, Serbia; vladimir.jovicic@med.bg.ac.rs; 5 Department of Medical Physiology, Faculty of Medicine, University of Belgrade, 11000 Belgrade, Serbia; nikola.topalovic@med.bg.ac.rs

**Keywords:** slug, snail, renal cell carcinoma, immunohistochemical markers, epithelial–mesenchymal transition

## Abstract

Renal cell carcinoma (RCC) is the deadliest urological neoplasm. Up to date, no validated biomarkers are included in clinical guidelines for the screening and follow up of patients suffering from RCC. Slug (Snail2) and Snail (Snail1) belong to the Snail superfamily of zinc finger transcriptional factors that take part in the epithelial–mesenchymal transition, a process important during embryogenesis but also involved in tumor progression. We examined Slug and Snail immunohistochemical expression in patients with different stages of renal cell carcinomas with the aim to investigate their potential role as staging and prognostic factors. A total of 166 samples of malignant renal cell neoplasms were analyzed using tissue microarray and immunohistochemistry. Slug and Snail expressions were evaluated qualitatively (presence or absence), in nuclear and cytoplasmic cell compartments and compared in relation to clinical parameters. The Kaplan–Meier survival analysis showed the impact of the sarcomatoid component and Slug expression on the survival longevity. Cox regression analysis separated Slug as the only independent prognostic factor (*p* = 0.046). The expression of Snail was associated with higher stages of the disease (*p* = 0.004), especially observing nuclear Snail expression (*p* < 0.001). All of the tumors that had metastasized showed nuclear immunoreactivity (*p* < 0.001). In clear cell RCC, we showed a significant relationship between a high nuclear grade and nuclear Snail expression (*p* = 0.039). Our results suggest that Slug and Snail could be useful immunohistochemical markers for staging and prognosis in patients suffering from various RCCs, representing potential targets for further therapy strategies of renal cancer.

## 1. Introduction

Renal cell carcinoma (RCC) is the deadliest urological neoplasm, accounting for approximately 2% of global cancer diagnoses and deaths, and is rapidly growing in incidence in the developed world [1,2]. This insidious neoplasm in most cases is found incidentally with magnetic resonance imaging (MRI), computed tomography (CT) scan, or ultrasound [2,3]. Only 10% of patients exhibit classic symptoms of hematuria, flank pain and palpable masses [3]. Clear cell renal carcinoma (ccRCC) is the most common histological subtype, followed by papillary (pRCC) and chromophobe (chRCC), as well as other rare malignant renal cell neoplasms such as collecting duct carcinoma (CDC) and Multilocular Cystic Renal Neoplasm of Low Malignant Potential (MCRNLMP) [2]. Despite new treatment options, RCC has a dismal late-stage, 5-year survival rate of only 12%. [2].

Many different clinical and epidemiological variables including age, gender, tumor–node–metastasis (TNM) stage and nuclear grade are used in routine medical practice, but they are of limited capacity to predict prognosis after surgical intervention or systemic therapy. This supports the need for developing new approaches and new prognostic factors in the management of RCC [4,5].

Up to date, no validated biomarkers are included in clinical guidelines for the screening and follow up of patients suffering from RCC [6]. Finding and approving such markers may be very useful in clinical practice by providing valuable insights into new cancer strategies with the aim of developing personalized treatments tailored to individual patients’ characteristics [6].

The epithelial-to-mesenchymal transition (EMT) is a highly conserved cellular program considered as a major driver in promoting tumor invasion and metastasis. In EMT, epithelial cells lose their epithelial properties, intercellular adhesion molecules and cytoskeletal-specific organization, and convert into a motile mesenchymal cell, characterized with invasive ability and metastatic capacity [7,8,9,10,11,12,13,14]. Growing evidence shows that the EMT program is involved in metastasis formation and therapy resistance in vivo, which can be at least partially responsible for poor prognosis in patients affected by cancer [10,12,15]. Starting from the fact that EMT in cancer tissues is commonly incomplete, reversible and transitional, the term partial EMT (pEMT) describes EMT in malignant cells more accurately [16,17].

Snail1 (Snail) and Snail2 (Slug) belong to the Snail superfamily of zinc finger transcriptional factors that take part in EMT, a process that is also important for normal morphogenesis [18]. Snail is required during embryonic development for mesoderm and neural crest formation. Slug also represses E-cadherin and induces a complete EMT. However, Slug binds with a lower affinity than Snail to the E-cadherin promoter [19,20]. In addition, a lot of data have indicated that Slug protein expression is increased in various cancer cells, including breast, ovarian, lung, pancreatic, and colorectal cancers [21].

Nuclear grade, tumor stage, vascular invasion, sarcomatoid transformation and fat-tissue invasion in clear cell RCC have been studied in several papers and correlate with disease prognosis and survival rate [22,23]. Besides this, different studies have analyzed the role of Slug and Snail in the progression of carcinomas, including RCC; however, results are contradicting and not conclusive [24]. Thus, further studies are necessary to clarify the role of the immunoexpression of these two markers and their utility in clinical practice [25].

In this study, we examined Slug and Snail immunohistochemical expression in patients with different renal cell neoplasms covering all disease stages, with the aim to investigate their potential role in disease progression, influencing the staging of the disease and patients’ survival.

## 2. Results

### 2.1. Evaluation of the Slug Expression and Its Influence on the Patients’ Survival

We analyzed 166 malignant renal cell neoplasms: Clear Cell Renal Cell Carcinoma, ccRCC (n = 108), low grade Papillary Renal Cell Carcinoma, pRCC—low grade (n = 7), high grade Papillary Renal Cell Carcinoma, pRCC—high grade (n = 15), Chromophobe Renal Cell Carcinoma, chRCC (n = 25), Collecting Duct Carcinoma (Bellini), CDC (n = 6), and Multilocular Cystic Renal Neoplasm of Low Malignant Potential, MCRNLMP (n = 5). The expression of Slug transcription factor was reported in 100 out of 166 tumors. An overview of clinical and pathological characteristics with regard to Slug expression in these tumors are presented in Table 1.

The frequency of Slug transcription factor expression was similar in males and females, and patients with Slug-positive and Slug-negative tumors did not differ in age of the subjects. We noticed that the tumors that expressed Slug had a slightly larger average size (median 65 mm) than tumors that did not show Slug immunopositivity (median 54 mm), but the observed difference was not statistically significant, Table 1. Gross examination revealed spreading into the perirenal adipose tissue and the renal vein in some cases; however, these features were not related to the Slug expression in renal cell neoplasms. On the other hand, spreading into the renal sinus was significantly related to the Slug positivity in tumors. Thus, Slug was detected in 94.7% of cases which were spread into the renal sinus, while in tumors that did not affect the renal sinus, the frequency of Slug expression was only 55.7% (*p* = 0.005).

**Figure 1:**
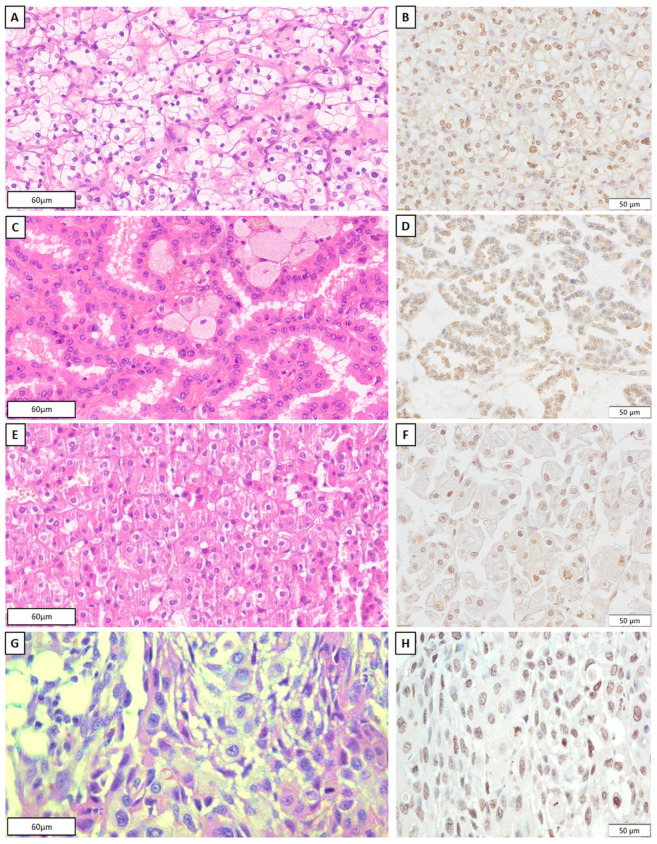
Morphology and immunohistochemical nuclear Slug expression in ccRCC (**A**—HE, and **B**—Slug), pRCC (**C**—HE and **D**—Slug), chRCC (**E**—HE and **F**—Slug), and CDC (**G**—HE and **H**—Slug).

In the various pathohistological types of malignant renal cell neoplasms the frequency of Slug immunopositivity ranged from 42.9% (pRCC) to 83.3% (CDC), but the differences were not statistically significant. Detection of nuclear Slug immunoexpression in different pathohistological tumor types is illustrated in Figure 1. Nevertheless, we observed a correlation between nuclear grade (NG) and nuclear expression of the Slug molecule by analyzing 108 ccRCCs. The nucleus of lower-grade tumors (I and II) were positive for Slug in 54.8% of cases, while the nucleus of higher-grade tumors (III and IV) showed positivity for Slug in as many as 82.1% (*p* = 0.001). Moreover, no correlation was observed between the stage of the disease and the expression of the Slug transcription factor. During the analysis, a statistically significant association was observed between Slug expression and the presence of a sarcomatoid component (*p* = 0.032). As many as 92.3% of tumors with a sarcomatoid component express Slug, while in tumors without a sarcomatoid component, only 60% showed Slug immunopositivity (Table 1).

Analysis of the Kaplan–Meier survival curve showed the influence of the sarcomatoid component and Slug expression on the length of survival. However, Cox regression analysis revealed Slug as the only independent prognostic factor (*p* = 0.046), Table 2.

**Figure 2:**
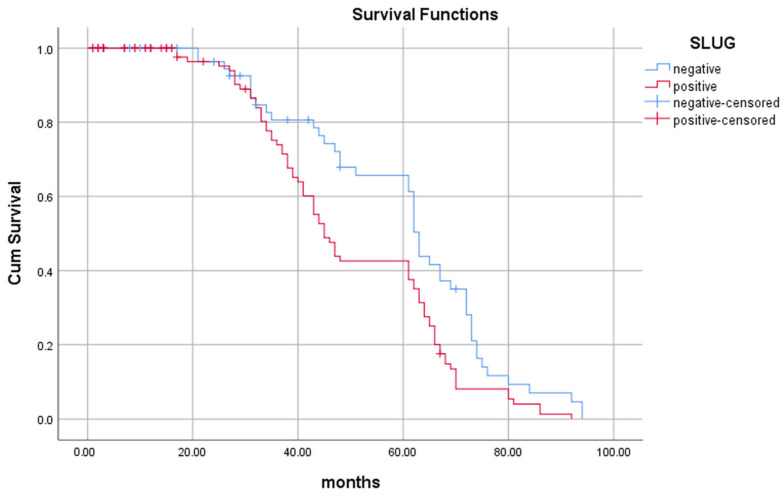
Length of survival of patients with Slug-positive (red line) and Slug-negative (blue line) tumors.

By analyzing the Kaplan–Meier survival curve, we noticed that during the first three years of follow-up there is no difference in survival in relation to Slug expression. However, after that period, the survival period is significantly longer in the group of patients whose tumors do not express Slug (*p* = 0.002) (Figure 2).

### 2.2. Evaluation of the Snail Expression and Its Influence on the Patients’ Survival

Snail molecule expression was recorded in 124 out of 166 tumors. Snail was observed in the cytosol and in the nucleus. A total of 72 tumors showed nuclear positivity, while cytoplasmatic positivity was observed in 124 tumors. A total of 61 tumors expressed Snail simultaneously in the nucleus and in the cytosol. The characteristics of the tumors are presented in Table 3.

The average size of Snail-positive tumors (70.5 mm) was slightly larger than the size of Snail-negative tumors (63.7 mm), but this difference was not statistically significant. We did not observe a significant difference in the frequency of expression of the Snail molecule in relation to the spread in the perirenal adipose tissue, renal vein and pelvic sinus (Table 3). However, tumors that invaded the renal vein significantly more often expressed Snail within the nucleus (*p* < 0.001).

**Figure 3:**
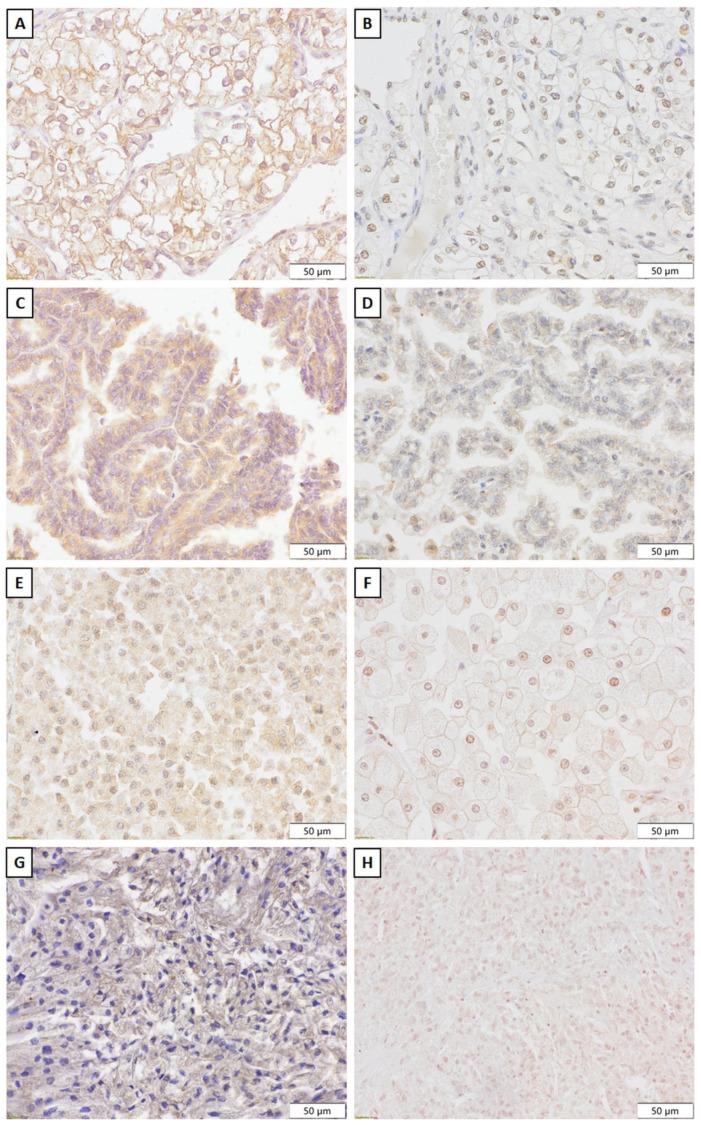
Immunohistochemical Snail expression in ccRCC (**A**—cytoplasmic, and **B**—nuclear), pRCC (**C**—cytoplasmic and **D**—nuclear), chRCC (**E**—cytoplasmic and **F**—nuclear), and CDC (**G**—cytoplasmic and **H**—nuclear).

In most pathohistological types, the frequency of Snail immunopositivity ranged from 57.1% to 93%, except for MCRNLMP, among which only 40% of tumors were positive for Snail. The observed difference was not confirmed statistically. Some tumors expressed Snail in the cytoplasm, while others exhibited nuclear Snail expression. Detection of Snail immunopositivity is illustrated in Figure 3.

Analyzing the association between the nuclear grade and nuclear expression of Snail, the majority of Snail-negative cases belonged to ccRCCs of lower nuclear grades (*p* = 0.041).

We did not observe a significant difference in the frequency of Snail molecule expression in relation to the presence of a sarcomatoid component.

However, it was observed that the increase in the frequency of Snail expression was accompanied by an increase in the stage of the disease (*p* = 0.003) (Table 3). All patients in the fourth stage of the disease had Snail-positive tumors. An association between nuclear localization and disease stage was also demonstrated (*p* < 0.001).

All (100%) metastasizing tumors were positive for Snail, while in tumors that did not metastasize, Snail positivity was recorded in 68% of cases (*p* = 0.033). In addition, all metastasizing tumors showed nuclear immunopositivity (*p* < 0.001).

**Figure 4:**
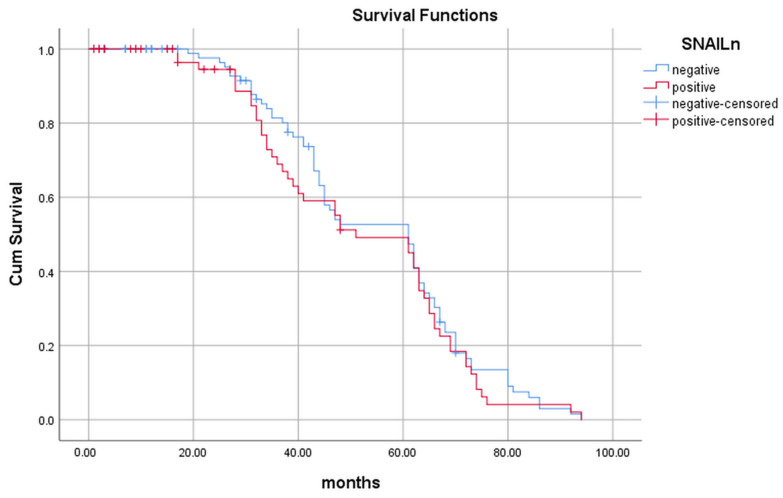
Length of survival of patients with nuclear Snail-positive (red line) and nuclear Snail-negative (blue line) tumors.

**Figure 5:**
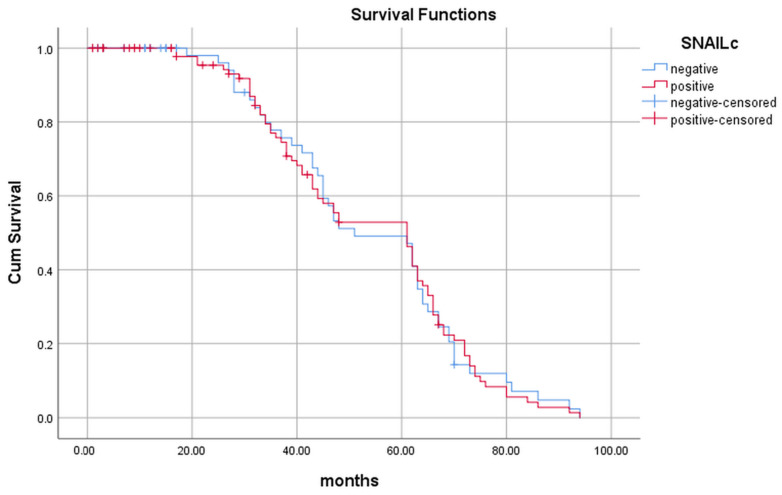
Length of survival of patients with cytoplasmic Snail-positive (red line) and cytoplasmic Snail-negative (blue line) tumors.

In patients whose tumors did not show nuclear Snail positivity, survival was significantly longer (47.3 ± 23 months) than in those whose tumors expressed Snail in the nucleus (39.3 ± 25), (*p* = 0.036), as shown in Table 3. Although we observed that patients whose tumors were not positive for Snail, either cytosolic or nuclear, lived longer than those whose tumors expressed Snail, Snail immunopositivity was not a predictive variable of patients’ survival, as shown by the Kaplan–Meier curve in Figure 4 (*p* = 0.493) and Figure 5 (*p* = 0.901).

## 3. Discussion

In the present study, a different pattern of immunohistochemical Snail expression was observed, regarding the stage of renal cancer and the length of survival. Snail immunopositivity was detected in both the nucleus and cytoplasm, but only nuclear expression correlated with a higher renal tumor stage, while the absence of nuclear Snail expression was accompanied with a significantly longer survival of renal cancer. It is assumed that Snail in the cytoplasm undergoes degradation by means of the ubiquitin-protease system, while Snail in the nucleus performs its biological function as a transcription factor [18].

On the other hand, we did not prove a correlation between the stage of the disease and Slug immunopositivity. Although when observing the first three stages, the frequency of Slug-positive tumors increases with the stage of the disease, the percentage of Slug-positive tumors in the fourth stage drops drastically. However, it was showed that Slug nuclear immunohistochemical expression was associated with the sarcomatoid component, as well as renal tumor survival. Carcinoma progression is linked to a partially dedifferentiated epithelial cell phenotype [26]. As already mentioned, Slug and Snail transcription factors were recognized as inductors of the epithelial–mesenchymal transition and metastasis [24]. Mesenchymal characteristics help cancer cells in metastatic process and tumor progression, including local invasion, intravasation into blood and lymphatic vessels, invading distant organs and tissues via circulation, and forming micrometastatic deposits [12,27]. In order to invade the extracellular matrix, tumor cells must separate from the surrounding cells, which they achieve by reducing the expression of E-cadherin, a molecule that is crucial for maintaining intercellular connections and the apical-basal polarization of cells. Many growth factors, including Slug and Snail protein, as well as environmental conditions, have been shown to induce EMT by increasing the activity of transcription factors responsible for downregulating E-cadherin, the hallmark protein of epithelial cells [28,29,30,31,32]. The increased expression of Slug and consequent reduction in E-cadherin expression has been observed in several neoplasms, including breast and ovarian carcinomas [21]. However, there are studies that challenge the importance of Slug during EMT. Mikami showed that the expression of Slug negatively correlates with the stage of RCC, and although some members of the Snail family are inducers of EMT, Slug did not affect this process [33]. On the contrary, Mystyk stated Slug as a prognostic factor, highlighting its role both during EMT and during cell migration [34]. In present study, we have shown that tumors with a sarcomatoid component express Slug significantly more often. Given that sarcomatoid differentiation is considered a morphological manifestation of the epithelial–mesenchymal transformation [35], this result supports the previously confirmed role of Slug molecules in the EMT. As the sarcomatoid component is thought to arise from the EMT, of which Snail is also main regulator [33,35], it is a little bit surprising that in present study no correlation between Snail expression and the presence of the sarcomatoid component of RCC was observed. However, although Snail expression has been shown to be more pronounced in tumors with a sarcomatoid component, tumors without this component also show increased Snail expression, which supports the theory that Snail contributes far more to the initiation of the EMT than to the morphological manifestations that are the results of this process [36]. In our study, over 70% of cancers showed Snail immunopositivity. Despite the lack of statistical significance, over half of the tumors with a sarcomatoid component expressed Snail in the nucleus, whereas the nuclei of over half of the tumors without a sarcomatoid component were negative for Snail.

Matrix metalloproteinases degrade the basement membrane and extracellular matrix, thus creating a pathway for tumor cell migration [37]. Members of the Snail family are recognized as inducers of various metalloproteinases. Working on squamous cell carcinoma cultures of the oral cavity, Huang proved that Slug induces membrane-type activity 4 matrix-metalloproteinases [38]. Although we observed that Slug-positive tumors penetrate more often into the renal sinus, we did not prove the connection between Slug and penetration into renal vein or into the perirenal fatty tissue. But we pointed out the association of Snail immunopositivity in kidney tumors with a higher stage of the disease and poor prognosis, what is similar to some already-published results [33]. In all patients with metastasis included in our research, we observed the expression of Snail, and presented that Snail is significantly more-often detected in RCCs that metastasize. However, Mikami and his colleagues did not prove the connection between the presence of Snail and metastasis. These differences can be explained by the fact that they made comparison based on the determined percentage of immunopositive cells, while the tumors were evaluated qualitatively in our cases. Besides this, a smaller number of patients was involved in their study compared to our research. In addition, we did not observe a significant difference regarding the average tumor size in relation to the presence of Snail. This is in accordance with the findings of Liu and Harada, who worked exclusively on organ-confined tumors, and independently found no connection between Snail transcription factor expression and tumor T stage [36,39]. These results suggest that this molecule does not affect local and regional tumor progression, but rather its metastatic tendency. Some works point out that Snail reduces the activity of Cyclin D1, necessary for the transition of the cell to the S phase [40]. This may possibly explain why, despite the greater metastatic tendency provided by Snail, the size and extension of the primary tumor mass are not greater in Snail-positive tumors. It can be assumed that metastatic tendency accompanied with Snail expression may be provoked by the increased activity of metalloproteinases and the reduced expression of adhesion molecules, as well as decreased expression of MUC1 (mucin 1, cell surface associated) protein, which contributes to increased cell mobility [41].

Nuclear grade is a reliable prognostic parameter in the histological analysis of ccRCC and pRCC [33,42]. Our work showed a correlation between nuclear Slug and Snail expression and nuclear grade in ccRCC. On the other hand, some authors reported that nuclear Snail expression was followed by higher nuclear grade in ccRCC [33], but found no correlation between Slug expression and the nuclear grade of ccRCC [33]. Andreina et al. stated that increased Snail expression was accompanied with a high nuclear grade, while Slug expression was followed with low nuclear grade. They concluded that the immunoexpression of Snail was significantly superior for advanced stages and Slug was overexpressed in early stages of ccRCC. [43]. In the study conducted by Zaldumbide et al., all cases of high-grade ccRCC presented Snail immunostaining and the negative immunoexpression was present only in low-grade cases [44]. Besides this, the results of Liu et al. showed that cytoplasmic Snail intensity correlates positively with nuclear grade. On the other hand, a high nuclear but not cytoplasmic Snail intensity indicates early recurrence and the poor survival of patients with localized ccRCC [36]. Examining whether Snail transcription factor expression is associated with the length of survival after diagnosis, we showed that both nuclear and total Snail expression were associated with shorter survival. This result is consistent with the result obtained by Lui [36] as well as with the results of similar studies in other types of cancer [45].

Slug expression is associated with a shorter survival in various tumor types [26,46]. Nevertheless, we have shown by analyzing the Kaplan–Meier survival curve that during the first three years of follow-up, there is no difference in the length of survival in relation to Slug expression transcription factor, but after that, the survival period becomes significantly longer in patients whose tumors do not express Slug. We also confirmed the significance of Slug as an independent prognostic factor. Moreover, in our research, the Kaplan–Meier curve analysis singled out Slug as a prognostic factor in papillary carcinomas, but Cox regression analysis indicated that NG was the only independent prognostic factor. Given the association between Slug expression and NG, it is likely that Slug affects prognosis indirectly, by increasing NG tumors.

The strength of the present work is the analysis of the subcellular localization of Snail and emphasizing the importance of its nuclear immunohistochemical expression regarding RCC stage and survival, and one of the main drawbacks is the relatively small number of patients and the unequal distribution of certain subtypes of kidney cancer. An additional limitation of this study is its retrospective nature, so the follow-up period is not long enough to allow us to reach definitive conclusions about certain questions. Therefore, further larger, multicentric, prospective studies are necessary to provide consistent findings regarding the potential significance of Slug and Snail expression as immunohistochemical markers for the staging and survival of RCC. For now, we can say that the results of the present study are promising.

## 4. Materials and Methods

### 4.1. Samples

The study included 166 patients who underwent surgery at the Clinic for Urology, Clinical Center of Serbia, between 2012 and 2018. By examining the medical documentation, data were collected from medical records and pathohistological reports. Information about the outcome, as well as time elapsed from nephrectomy to death caused by the tumor, was obtained from the records of the Institute for Public Health “Dr. Milan Jovanovic Batut”. If fatal outcome did not occur, the survival period was represented by the time from nephrectomy to the last check-up at the Urology Clinic.

### 4.2. Pathological Evaluation and Ethics

The tumor diagnosis was confirmed by biopsy at the Institute of Pathology, Faculty of Medicine, University of Belgrade. The analysis of the hematoxylin-eosin preparations determined the type of tumor, nuclear grade (only for ccRCC) and the presence of a sarcomatoid component. The stage of the tumor was determined by analyzing the operative material and examination of the preparation, in accordance with TNM system [47]. The material was collected from the archive of Pathology Department, Clinic of Urology, University Clinical Center of Serbia, Belgrade. The Ethic Committee of the Clinic of Urology of Clinical Center of Serbia granted approval to collect the samples from the archive, and carry out the study (Application Ref: 0152/20). The study presented here was conducted following all ethical standards laid down in the 1964 Declaration of Helsinki. Since the study was carried out retrospectively, according to our ethical issues, informed consents of patients were not required.

### 4.3. Tissue Microarray

From the paraffin molds, tissue cylinders were taken to make a tissue microarray, and sampling was performed from the region of interest, in triplicate, using a 0.6 mm hollow medical needle. The tissue cylinders taken were then embedded in a paraffin block and precisely arranged in an array. Paraffin blocks of the tissue microarray were cut in the microtome, to a thickness of 5 µm and placed on microscopic slides, which were further used for immunohistochemical analysis.

### 4.4. Immunohistochemistry

Immunohistochemistry was performed on tissue microarray slides. After deparaffinization in xylene and hydration, the slides were placed in citrate buffer (pH 6.0) and exposed to microwaves for 20 min at 400 W. Peroxidase activity was blocked with 1% BSA (bovine serum albumin). After antigen retrieval, incubation with primary antibodies Slug (1:100, ab27568, Abcam, Boston, MA, USA) and Snail (1:100, PA5-11923, Thermo Fischer Scientific, Waltham, MA, USA) was performed for 1 hour. EnVision^TM^ (DAKO, Copenhagen, Denmark) was used to visualize the antigen–antibody reaction with 3,3′-diaminobenzine (DAB) and subsequent contrast with hemalaun (Merz, WI, USA). Negative controls were obtained by omitting the primary antibody. Slides were examined using a BX53 light microscope with a DP12CCD camera (Olymus, Hamburg, Germany).

Immunostaining of both markers was independently evaluated by three pathologists (M.Z., G.N., D.D), who were blinded to the patient outcome and pathological information. Valid immunoreactivity was considered as follows: nuclear staining of Slug and Snail, as well as cytoplasmic staining along with nuclear immunopositivity. Tumors were considered to be positive when at least one tumor cylinder in TMA was positive, with more than 50% positive tumor cells, and with at least moderate intensity of the staining. A consensus was achieved for all samples.

### 4.5. Statistical Analysis

The statistical analysis was performed using IBM SPSS software, version 20.0, using appropriate statistical tests. Each numerical variable was tested for normality of distribution, using Shapiro–Wilk and Kolmogorov–Smirnov tests, as well as considering skewness and kurtosis, before the implementation of other statistical tests. Numerical variables with normal distributions were analyzed using the Student t-test, while numerical data without normal distributions were analyzed applying the Mann–Whitney U test. Nominal data were analyzed using the χ2 test or with a Mann–Whitney U test and Kruskal–Wallis test, which was also used for ordinal data. Demographic, clinical and pathohistological characteristics of kidney tumors were examined in relation to the presence and localization of the Slug and Snail molecules. *p* values < 0.05 were considered to be significant.

All data recorded at the time of biopsy (demographic, clinical and pathohistological), including the Slug and Snail immunohistochemical expressions, represented potential predictive factors. Univariate analysis was performed using the Kaplan–Meier estimator in order to identify variables significantly associated with lethal outcome. Differences between two groups of patients (with and without adverse outcome) were assessed by two-sided log rank test. In univariate analysis, potential predictors of kidney dysfunction development were identified using a significance value of *p* < 0.05. These potential predictors were then included in a multivariate Cox’s regression model, which was applied to identify variables that were independently associated with lethal outcome.

## 5. Conclusions

Slug is more frequently expressed in tumors with a sarcomatoid component, which confirms its role in the EMT process. The survival period is significantly longer in patients whose tumors do not express Slug. Our results show the correlation of Snail expression, especially nuclear expression, with higher stages of kidney tumors, a higher degree of invasiveness of the tumor themselves, and shorter survival. Our results suggest that Slug and Snail may be useful immunohistochemical markers for staging and prognosis in patients suffering from various RCCs, representing potential targets for further therapy strategies of renal cancer.

## Figures and Tables

**Table 1:** Clinical and pathological features of renal cell carcinoma with regard to Slug transcription factor expression.

Slug			
	Positive (n = 100)	Negative (n = 66)	*p* Value
Age (years)			
	59.9 ± 11.0	62.7 ± 10.6	0.129
Gender			
Male	33 (58.9%)	23 (37.7%)	0.908
Female	67 (60.9%)	43 (39.1%)	
Tumor size (mm)			
	65 (10–340)	54 (20–180)	0.145
Invasion in adipose tissue			
Yes	11 (55%)	9 (45%)	0.172
No	89 (71.8%)	35 (28.2%)	
Penetration to renal sinus			
Yes	18 (94.7%)	1 (5.3%)	0.005 *
No	82 (55.7%)	65 (44.3%)	
Permeation to renal vein			
Yes	14 (67.1%)	6 (32.9%)	0.085
No	86 (58.9%)	60 (41.1%)	
Tumor type			
ccRCC	62 (57.4%)	46 (42.6%)	0.712
pRCC—low grade	3 (42.9%)	4 (57.1%)	
pRCC—high grade	9 (60%)	6 (40%)	
chRCC	18 (72%)	7 (28%)	
CDC—Bellini	5 (83.3%)	1 (16.7%)	
MCRNLMP	3 (60%)	2 (40%)	
Nuclear grade (only ccRCC)			
I and II	37 (48%)	40 (52%)	0.001 *
III and IV	25 (80.6%)	6 (19.4%)	
Sarcomatoid component			
Yes	13 (92.3%)	1 (7.7%)	0.032 *
No	87 (57.2%)	65 (42.8%)	
TNM stage			
I	28 (56%)	22 (44%)	0.208
II	11 (61.1%)	7 (38.9%)	
III	40 (75.5%)	13 (24.5%)	
IV	6 (54.5%)	5 (45.5%)	
Metastasis			
Yes	6 (54.5%)	5 (45.5%)	0.536
No	94 (60.6%)	61 (39.4%)	
Outcome			
Survived	76 (60.8%)	49 (39.2%)	0.574
Died	24 (58.5%)	17 (41.5%)	

ccRCC—Clear Cell Renal Cell Carcinoma. low grade pRCC—low grade Papillary Renal Cell Carcinoma. high grade pRCC—high grade Papillary Renal Cell Carcinoma. chRCC—Chromophobe Renal Cell Carcinoma. CDC (Bellini)—Collecting Duct Carcinoma (Bellini). MCRNLMP—Multilocular Cystic Renal Neoplasm od Low Malignant Potential. * *p* < 0.05.

**Table 2:** The results obtained by the Kaplan–Meier survival curve and Cox regression analysis, * *p* < 0.05.

	Kaplan Maier Univariant Analysis		Cox regression Multivariant Analysis	
Prognostic Factor	Average—Months	*p* Value	Hazard Ratio	*p* Value
	(95%CI)		(95% CI)	
Gender				
Female	48 (43–66)	0.251		
Male	61 (45–62)			
Invasion to perirenal adipose tissue				
Yes	61 (46–63)	0.384		
No	65 (43–70)			
Penetration to renal sinus				
Yes	45 (41–64)	0.411		
No	62 (61–64)			
Permeation to renal vein				
Yes	63 (35–64)	0.446		
No	61 (47–63)			
Sarcomatoid component				
Yes	39 (33–47)	0.032 *	2.19 (0.87–5.54)	0.097
No	61 (47–63)			
TNM staging				
I and II	62 (47–64)	0.180		
III and IV	45 (38–62)			
Slug expression				
Yes	45 (41–64)	0.002 *	1.73 (1.16–2.57)	0.046 *
No	63 (62–72)			
Metastasis				
Yes	41 (41-NA)	0.956		
No	61 (45–62)			

**Table 3:** Clinical and pathohistological features of malignant renal cell neoplasms with regard to Snail transcription factor expression.

Snail Nuclear Expression				Snail Cytoplasmatic Expression		
	Positive (n = 72)	Negative (n = 94)	*p* Value	Positive (n = 124)	Negative (n = 42)	*p* Value
Tumor size (mm)						
	71.8 ± 47.6	66.4 ± 30.6	0.416	70.5 ± 41.6	63.7 ± 29.1	0.251
Penetration to renal sinus						
Yes	10 (52.3%)	9 (47.4%)	0.462	17 (90.0%)	2 (10.0%)	0.090
No	62 (42.2%)	85 (57.8%)		107 (72.8%)	40 (27.2%)	
Permeation to renal vein						
Yes	16 (80.0%)	4 (20.0%)	<0.001	18 (90.0%)	2 (10.0%)	0.090
No	56 (38.3%)	90 (61.7%)		106 (72.6%)	40 (27.4%)	
Invasion to perirenal adipose tissue						
Yes	13 (65.0%)	7 (35.0%)	0.051	18 (90.0%)	2 (10%)	0.090
No	59 (40.4%)	87 (59.6%)		106 (72.6%)	40 (27.4%)	
Tumor type						
ccRCC	49 (45.4%)	59 (54.6%)	0.467	80 (74.1%)	28 (25.9%)	0.143
pRCC—low grade	2 (28.6%)	5 (71.4%)		4 (57.1%)	3 (42.9%)	
pRCC—high grade	4 (26.7%)	11 (73.3%)		14 (93.3%)	1 (6.7%)	
chRCC	13 (52.0%)	12 (48.0%)		20 (80.0%)	5 (20.0%)	
CDC (Bellini)	3 (50.0%)	3 (50.0%)		4 (66.7%)	2 (33.3%)	
MCRNLMP	1 (20.0%)	4 (80.0%)		2 (60.0%)	3 (40.0%)	
Nuclear grade (only ccRCC)						
I	11 (57.9%)	8 (42.1%)	0.041 *	16 (84.2%)	3 (15.8%)	0.343
II	20 (33.9%)	39 (66.1%)		40 (67.8%)	19 (32.2%)	
III	11 (68.8%)	5 (31.2%)		14 (87.5%)	2 (12.5%)	
IV	7 (53.8%)	6 (46.2%)		10 (76.9%)	3 (23.1%)	
Sarcomatoid component						
Yes	9 (64.3%)	5 (35.7%)	0.160	10 (71.4%)	4 (28.6%)	0.756
No	63 (41.4%)	89 (58.6%)		114 (75.0%)	38 (25.0%)	
TNM stage						
I	15 (27.8%)	39 (72.2%)	<0.001	31 (57.4%)	23 (42.6%)	0.003 *
II	6 (33.3%)	12 (66.7%)		11 (61.1%)	7 (38.9%)	
III	25 (46.3%)	29 (53.7%)		44 (81.5%)	10 (18.5%)	
IV	11 (100%)	0 (0%)		11 (100%)	0 (0%)	
Survival (months)						
	39.3 ± 25.4	47.3 ± 23.0	0.037 *	42.2 ±24.4	48.4 ± 23.7	0.160

ccRCC—Clear Cell Renal Cell Carcinoma. low grade pRCC—low grade Papillary Renal Cell Carcinoma. high grade pRCC—high grade Papillary Renal Cell Carcinoma. chRCC—Chromophobe Renal Cell Carcinoma. CDC (Bellini)—Collecting Duct Carcinoma (Bellini). MCRNLMP—Multilocular Cystic Renal Neoplasm od Low Malignant Potential. * *p* < 0.05.

## Data Availability

The data presented in this study are available in article.
